# Identification of Sanguinarine Metabolites in Rats Using UPLC-Q-TOF-MS/MS

**DOI:** 10.3390/molecules28227641

**Published:** 2023-11-17

**Authors:** Mengting Liu, Zhiqin Liu, Zhuang Dong, Xianglin Zou, Jianguo Zeng, Zihui Yang

**Affiliations:** 1 Hunan Key Laboratory of Traditional Chinese Veterinary Medicine, Hunan Agricultural University, Changsha 410128, China; lmt19970808@163.com (M.L.); lzq.221338@stu.hunau.edu.cn (Z.L.); dzlebron0701@163.com (Z.D.); zxl19991113@163.com (X.Z.); 2 College of Veterinary Medicine, Hunan Agricultural University, Changsha 410128, China

**Keywords:** sanguinarine, UPLC-Q-TOF-MS/MS, metabolites, rats, in vivo

## Abstract

Sanguinarine (SAN), as the main active component of a traditional Chinese veterinary medicine, has been widely used in the animal husbandry and breeding industry. However, the metabolites of SA are still uncertain. Therefore, this research aimed to investigate the metabolites of SA based on rats in vivo. The blood, feces, and urine of rats were collected after the oral administration of 40 mg/kg SAN. Ultra-high-performance liquid chromatography coupled with quadrupole time-of-flight mass spectrometry (UPLC-Q-TOF-MS/MS) was employed to identify the metabolites of SAN. The elemental composition of sanguinarine metabolites was inferred by analyzing their exact molecular weight, and the structures of the metabolites were predicted based on their fragment ions and cleavage pathways. A total of 12 metabolites were identified, including three metabolites in the plasma, four in the urine, and nine in the feces. According to the possible metabolic pathways deduced in this study, SAN was mainly metabolized through reduction, oxidation, demethylation, hydroxylation, and glucuronidation. This present research has summarized the metabolism of SAN in rats, which is helpful for further studying the metabolic mechanism of SAN in vivo and in vitro.

## 1. Introduction

Sanguinarine (SAN), a benzophenanthridine alkaloid, is the main component of *Macleaya cordata* (Willd.) R. Br. (English name: Plume Poppy), from the poppy family. It has been demonstrated that SAN possesses multiple biological activities, in particular antibacterial and anti-inflammatory properties, as well as regulating intestinal flora [1,2]. As a result of antibiotic growth promoters (AGPs) being banned in China, AGPs have been completely withdrawn from the market [3]. Meanwhile, traditional Chinese veterinary medicines have become the focus for replacing AGPs. In China, SAN has been successfully developed into a traditional Chinese veterinary medicine for its outstanding growth-promoting effect [4,5,6]. Moreover, it has been authorized for long-term addition to feed and has been widely used in the breeding industry [7].

Even though there are many reports on the growth promoting mechanism [6] and pharmacological activities [8,9] of SAN, studies on the metabolites of SAN are still limited. In the last century it was reported that SAN can produce toxic effects in rats [10]; furthermore, SAN is a major active substance in *Argemone mexicana* seed oil (AO), which caused “epidemic dropsy” in New Delhi, India [11]. Moreover, numerous studies have shown evidence of a correlation between AO and genotoxicity [12,13]. In addition, SAN also leads to severe cardiac abnormalities and dysfunction; recently, decreases in the heart rate and red blood cell count of zebrafish were reported [14]. It was found that 14,000 mg/kg Sangrovit (SAN as active substance) resulted in an elevated reduction in glutathione level and superoxide dismutase activity in the liver [15]. *Macleaya Cordata* extract, of which SAN is a major ingredient, was found to exhibit hypotoxic properties after acute exposure in SD rats and ICR mice [16]. As it is well known, drug metabolism is closely related to drug bioactivities and toxicity. Therefore, the identification of drug metabolites is of vital importance for elucidating the drug’s pharmacological mechanisms and pharmacokinetic behavior.

The earliest research showed that 3,4-benzoacridine was the metabolite of SAN [17]; nevertheless, a later study reported that SAN was metabolized to dihydrosanguinarine (DHSAN) [18], which was identified in the plasma and livers of rats using high-performance liquid chromatography electrospray mass spectrometry (HPLC/ESI-MS). The formation of DHSAN might be the first step of SAN detoxification in an organism and its subsequent elimination in phase II reactions. However, benz[c]acridine (BCA), which was reported as the metabolite of SAN in the literature, was found neither in the urine nor in the plasma or liver [19]. Another study reported that there were six metabolites formed in rat liver microsomes (RLM); the main metabolite (*m*/*z* 320) resulted from ring cleavage of SAN followed by demethylation, whereas the metabolite (*m*/*z* 348) was oxidized by recombinant human cytochrome P450 (CYP) in the presence of NADPH. The diol-sanguinarine metabolite (*m*/*z* 366) formed by RLM is derived from a putative epoxy-sanguinarine metabolite (*m*/*z* 348). 5,6-Dihydrosanguinarine is the prominent derivative formed from SAN in cells that do not express CYP [20].

Interestingly, the formation of the dihydro metabolite has been reported in the literature, which could be the main route of biotransformation (detoxification) of the benzo[c]phenanthridines in human hepatocytes. In addition, demethylation occurred on DHSAN in human hepatocytes, pig liver microsomes, and cytoplasms [21,22]. Zhang et al.’s [22] study showed that seven metabolites were identified in pig liver preparations; DHSAN was the main metabolite formed in the liver microsomes and in the cytosol. One oxidative metabolite and two O-demethylenated metabolites of SAN (*m*/*z* 320) were found in the trichloroacetic-acid-treated microsomal samples. However, a SAN pseudo base and two additional O-demethylenated metabolites of DHSAN (*m*/*z* 322) were found only in the acetonitrile-treated microsomal samples. Moreover, chelerythrine (CHE, an analogue of SAN) has been reported in many metabolites in rat vivo or vitro; the results show that the biotransformation pathways of CHE include reduction, demethylation, hydroxylation, and methylene dioxy cycle opening. Glucuronidation mainly occurred in the side chain of the benzophenanthridine parent structure; the metabolites of CHE in phase I and phase II were 25 and 8, respectively [23].

Enzymes are essential for metabolic reactions in organisms. The inhibitory effect of SAN on the cytochrome P450 enzyme has been reported, especially in CYP1A2, CYP2C9, CYP2C8, and CYP3A4, while it is not obvious in CYP2A6, CYP2D6, and CYP2E1 (Qi et al., 2013) [24]. Zhang confirmed that benzoquinone oxidase (NQO1) was employed in the reduction in SAN’s imine bond and DHSAN was generated by reduction with less toxicity than the metabolite in CYP1A1-metabolizing pathway [25]. All in all, the metabolism of SAN has been preliminarily studied by many researchers, but all of the studies were based on the liver microsomes in pigs, rats, and humans. There is no research on the metabolism of SAN in rats in vivo.

The aim of this study was to identify the metabolites of SAN in rats via UPLC-Q-TOF-MS/MS, and to infer the possible metabolic pathways so as to provide a research basis for the future studies of the metabolic mechanism of SAN in animals and the key enzymes in each metabolic pathway.

## 2. Results and Discussion

### 2.1. Fragmentations of Precursor Compound

**Figure 1:**
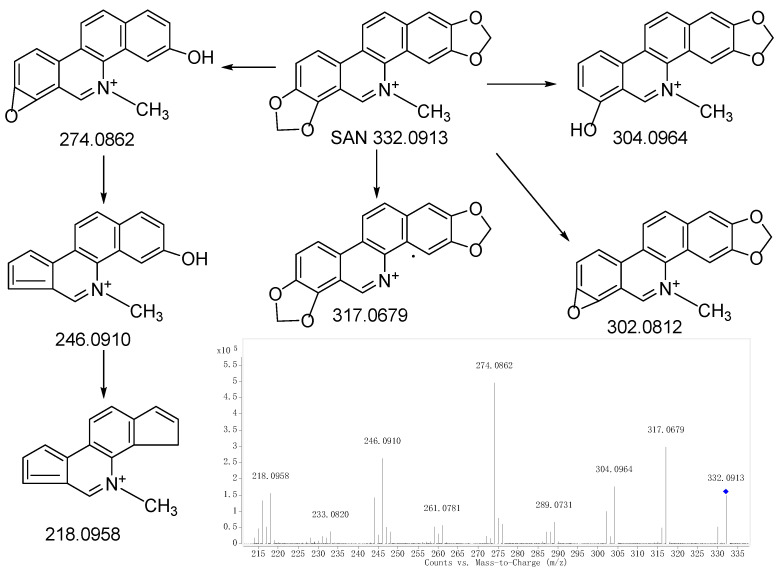
MS/MS spectrum of SAN and the proposed fragmentation pathways.“◆”: precursor ion.

Comparing the fragment ions of metabolites with the parent drug is a common and effective measure to identify the metabolites. In order to acquire an accurate identification, it is necessary to first find the fragmentation of the parent drug. Combined with the fragmentations of SAN mentioned in the literature [26], the fragmentations of SAN are shown in Figure 1. The retention time, elemental composition, type of reaction, accurate mass, and mass error are shown in Table 1. Twelve metabolites were finally identified, with three metabolites in the plasma (M1, M2, and M9), four metabolites in the urine (M1, M7, M11, and M12), and nine metabolites (M1−M8 and M10) in the feces. All of the metabolite structures are shown in Figure S1.

Rat liver microsomes are most commonly used in metabolic research. The results of rat liver microsomes often provide important references for the research of in vivo of rats. According to the results reported by Deroussent et al. [20], only six metabolites of SAN had been identified, half of what was found in this research. Obviously, there is a significant difference between in vitro and in vivo metabolism research.

### 2.2. Structural Elucidation

**Figure 2:**
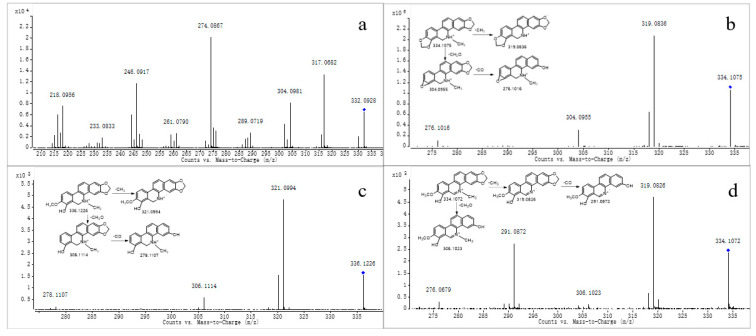
MS/MS spectra of M0 (**a**), M1 (**b**), M2 (**c**), and M3 (**d**). “◆”: precursor ion.

Metabolite M0 produced the molecular ion M^+^ at *m*/*z* 332.0928, with a retention time of 13.496 min, which is in accordance with the molecular formula of C_20_H_15_NO_4_ (calculated for C_20_H_15_NO_4_^+^: 332.0917). The MS/MS spectrum (Figure 2a) displayed the precursor ion at *m*/*z* 332.0928, which generated the product ion at *m*/*z* 317.0682, 304.0981, 302.0810, and 274.0867 through the loss of CH_3_, CO, CH_2_O, and C_2_H_2_O_2_, respectively, and *m*/*z* 274.0867 further produced *m*/*z* 246.0917 and 218.0956 through the loss CO and 2CO, respectively. The product ions and fragmentation patterns were the same as SAN, and M0 was further confirmed using the reference standard. Under the same LC/MS condition, M0 showed the same chromatographic retention time, accurate mass, and product ions as the reference standard of SAN. Therefore, M0 was unambiguously identified as SAN.

Metabolite M1 produced the quasi-molecular ion [M+H]^+^ at *m*/*z* 334.1075 with a retention time of 24.281 min, which was consistent with the molecular formula of C_20_H_15_NO_4_ (calculated for [M+H]^+^: 334.1074). The fragment ion at *m*/*z* 334.1075 generated fragments at *m*/*z* 319.0836, 304.0955, and 276.1016 through the loss of CH_3_, CH_2_O, and C_2_H_2_O_2_, respectively (Figure 2b). The product ions mentioned above were 2 Da more than that of M0, which was consistent with the fragmentation pathways of M0; we suggest it could be a reductive metabolite of M0. M1 was further confirmed through comparison with the reference standard. Using the same LC-MS condition, M1 showed the same retention time, accurate mass, and fragment ions through comparison of the standard of DHSAN. Therefore, M1 was identified as DHSAN.

Metabolite M2 produced the quasi-molecular ion [M+H]^+^ at *m*/*z* 336.1226 with a retention time of 19.730 min, which was in accordance with the molecular formula of C_20_H_17_NO_4_ (calculated for [M+H]^+^: 336.1230). The precursor ion at *m*/*z* 336.1226 generated a product ion at *m*/*z* 321.0994, 306.1114, and 278.1107 through the loss of CH_3_, CH_2_O, and C_2_H_2_O_2_, respectively, as observed in the MS/MS spectrum (Figure 2c). All of the product ions were 2 Da more than that of DHSAN, which agreed with the fragmentation pathways of DHSAN. Deroussent et al. also found this metabolite in the rat liver microsomes [20]. So, we suggest it could be the product of DHSAN after the ring-cleavage reaction.

Metabolite M3 produced the molecular ion M^+^ at *m*/*z* 334.1072 (Rt = 12.098 min), which was in accordance with the molecular formula of C_20_H_16_NO_4_ (calculated for [M]^+^: 334.1074). The MS/MS spectrum (Figure 2d) demonstrated that the precursor ion at *m*/*z* 334.1072 generated the product ion at *m*/*z* 319.0826 through the loss of CH_3_, and *m*/*z* 291.0872 was produced from the ion at *m*/*z* 319.0826 through the loss of CO. The above fragmentation pathways agreed with the structural characteristics of M2. On the other hand, M3 had the same fragment ions as demethylation chelerythrine, as reported by Q. Lin et al. [23]. Therefore, we speculate it could be a product ion of M2 after oxidation.

**Figure 3:**
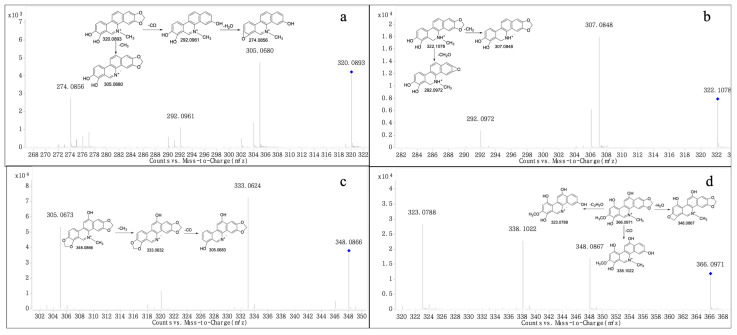
MS/MS spectra of M4-5 (**a**), M6 (**b**), M7 (**c**), and M8 (**d**). “◆”: precursor ion.

Metabolites M4 and M5 produced the molecular ion M^+^ at *m*/*z* 320.0893 and 320.0912, respectively, with a retention time of 11.014 and 12.721 min, respectively, which were in accordance with the molecular formula of C_19_H_14_NO_4_ (calculated for [M]^+^: 320.0917). The MS/MS spectrum (Figure 3a) showed the precursor ion generated the product ion at *m*/*z* 305.0680 and 292.0961 through the loss of CH_3_ and CO, and *m*/*z* 292.0961 further produced *m*/*z* 274.0856 through the loss of H_2_O. The product ions of M4 and M5 were 14 Da less than M3, and were consistent with the fragmentation pathway of M3; thus, we suggest they could be products of M3 after demethylation, as both M4 and M5 were isomers.

Metabolite M6 produced the quasi-molecular ion [M+H]^+^ at *m*/*z* 332.1078 with a retention time of 16.749 min, which was in accordance with the molecular formula of C_19_H_15_NO_4_ (calculated for [M+H]^+^: 332.1074). The MS/MS spectrum (Figure 3b) showed that the precursor ion at *m*/*z* 332.1078 generated a product ion at *m*/*z* 307.0848 and 292.0972 through the loss of CH_3_ and CH_2_O, respectively. The product ions all were 14 Da less than M2, based on the fragmentation ions found; we suggest that M6 could be the product of M2 after demethylation.

Metabolite M7 produced the molecular ion M^+^ at *m*/*z* 348.0866 (RT = 16.749 min), which was consistent with the molecular formula of C_20_H_14_NO_5_ (calculated for [M]^+^: 348.0866). The fragment at *m*/*z* 333.0624 was generated by the precursor ion at *m*/*z* 348.0866 through the loss of CH_3_, and the product ion at *m*/*z* 305.0673 was formed from the ion at *m*/*z* 348.0866 through the loss of CO. The above behaviors were observed in the MS/MS spectrum (Figure 3c). The product ions all were 16 Da more than M0. We suggest that M7 could be a product of M0 after hydroxylation; however, the position of hydroxylation was uncertain.

The fragment ion at *m*/*z* 366.0971 (RT = 17.864 min) was formed from Metabolite M8, which was in accordance with the molecular formula of C_20_H_16_NO_6_ (calculated for[M]^+^: 366.0972). The MS/MS spectrum (Figure 3d) demonstrated the precursor ion at *m*/*z* 366.0971 generated the product ion at *m*/*z* 348.0867, 338.1022 and 323.0788 by loss of H_2_O, CO and C_2_H_3_O, All the product ions were 32 Da more than M3, we suggested that M8 could be the product of M3 after hydroxylation and the hydrogen in the benzene ring was doubly hydroxylated. However, the position of hydroxylation was uncertain.

**Figure 4:**
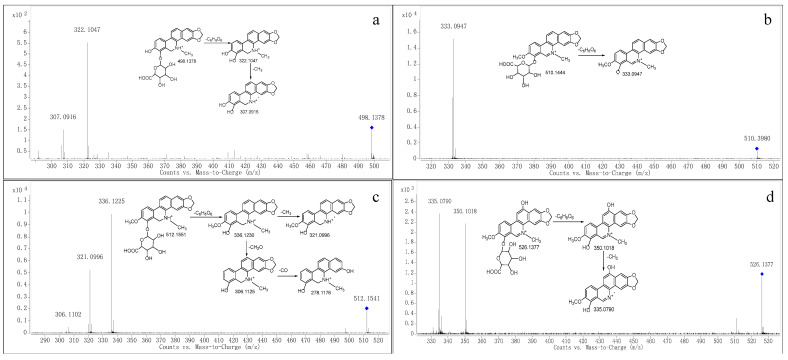
MS/MS spectra of M9 (**a**), M10 (**b**), M11 (**c**) and M12 (**d**). “◆”: precursor ion.

Metabolite M9 was eluted at a retention time of 19.730 min with the quasi-molecular ion [M+H]^+^ at *m*/*z* 498.1398. The elemental composition was proposed to be C_25_H_23_NO_10_ (calculated for [M+H]^+^: 498.1395), which demonstrated that M9 was glucuronide conjugate of M6. The MS/MS spectrum (Figure 4a) showed two characteristic product ions at *m*/*z* 322.1047 and 307.0916, which were in accordance with those of M6. Therefore, M9 was proposed as an acyl glucuronide conjugate of M6.

Metabolite M10 produced the molecular ion M^+^ at *m*/*z* 510.1444 (RT = 23.847 min), which was in accordance with the molecular formula of C_26_H_24_NO_10_ (calculated for [M]^+^: 510.1395), which indicated that M10 was a glucuronide conjugate of M3. The MS/MS spectrum (Figure 4b) showed the fragment ion at *m*/*z* 333.0947, which was consistent with M3. Therefore, M10 was tentatively identified as a glucuronide conjugate of M3.

Metabolite M11 produced the quasi-molecular ion [M+H]^+^ at *m*/*z* 512.1541 (RT = 15.213 min), which was in accordance with the molecular formula of C_26_H_25_NO_10_ (calculated for [M+H]^+^: 512.1551). The MS/MS spectrum (Figure 4c) showed that M2 was generated from the ion at *m*/*z* 512.1541 through the loss of C_6_H_8_O_6_, and *m*/*z* 321.0996 and 306.1102 were formed from M2 through the loss of CH_3_ and CH_2_O, respectively. Therefore, we suggest it could be a product of M2 because of glucuronidation.

The *m*/*z* at 526.1377 (RT = 15.691 min) was produced from Metabolite M12, whose fragmentation pathways were in accordance with the molecular formula of C_26_H_24_NO_11_ (calculated for [M]^+^: 526.1344). The MS/MS spectrum (Figure 4d) showed that the precursor ion at *m*/*z* 526.1377 generated the product ion at *m*/*z* 350.1018 through the loss of C_6_H_8_O_6_. Moreover, the fragment at *m*/*z* 335.0790 was produced from *m*/*z* at 350.1018 through the loss of CH_3_. Therefore, we suggest M12 could be the product of glucuronidation of M3 after hydroxylation.

### 2.3. Metabolite Profile and Metabolic Pathways of SAN

**Figure 5:**
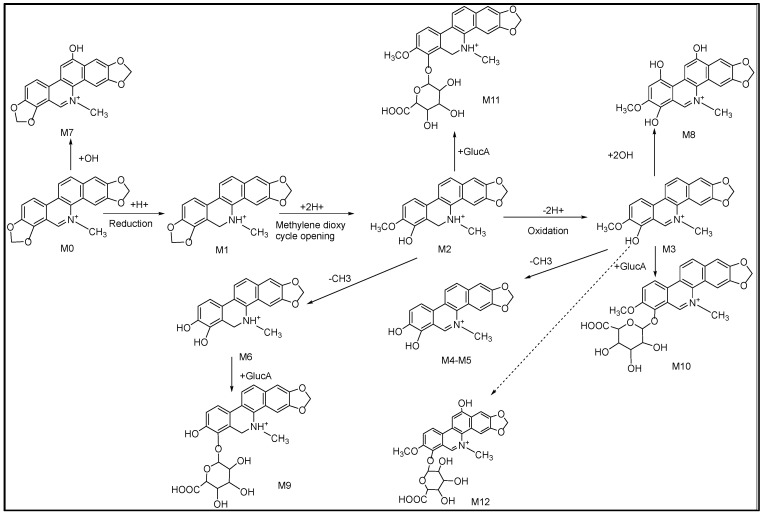
Proposed metabolic pathways of SAN in rats.

According to the fragmentations of SAN mentioned above, based on both the inferred metabolite structures and the document literature, the possible metabolic pathways are presented in Figure 5. Considering that the number of metabolites in the plasma and urine was much less than that in the feces, a possible reason is that SAN was orally administrated to the rats, which led to a low dose absorption into the blood. The previous study reported that after a single oral dose of ^3^H-sanguinarine, more than 42% of the ingested radioactivity was present in the gastrointestinal tract [27].

A HPLC-MS/MS method was established for the simultaneous quantitative analysis of SAN and DHSAN in chicken plasma, and was applied to a pharmacokinetic study in chicken. The results showed that C_max_ (0.9 ng/mL) of SAN was attained at 0.38 h and C_max_ (5.17 ng/mL) of DHSAN was attained at 0.25 h, which indicated that both SAN and DHSAN were rapidly absorbed in the chickens; even though the absorbed dose of SAN was low, DHSAN was more easily absorbed [28]. The pharmacokinetics results showed that after SAN was administered through subcutaneous injection, the C_max_ of SAN (30.16 ng/mL) and DHSAN (5.61 ng/mL) were achieved at 0.25 h; meanwhile, after the oral administration of SAN, C_max_ of SAN (3.40 ng/mL) and DHSAN (2.41 ng/mL) were attained at 2.75 h, and the relative bioavailability of SAN was lower than DHSAN, which indicated that SAN had difficulty being absorbed after oral administration in pigs [29]. Moreover, male and female rats were intragastrically administered SAN (0.5 mg/kg body weight and 5 mg/kg body weight dose for 28 days), and the C_max_ of SAN (low dose) in female and male rats was reached (4.76 and 3.88 ng/mL) at 2.17 h (T_max_) and the C_max_ of SAN (high dose) in female and male rates was reached (33.12 and 24.36 ng/mL) at 2.17 h (T_max_) [30]. There were a large amount of SAN excreted from the feces after SAN was orally administered [31]. In conclusion, all of the studies mentioned above indicate that the absorption of SAN in vivo was poor, which could be the main reason for the differences in the amount of metabolites produced in the plasma, urine, and feces.

There were different results found for the metabolite of SAN between this study and other studies in the literatures. A metabolite with *m*/*z* 350.1023 of SAN was identified by Zhang et al. in pig liver microsomes; however, this was not included in the present research, and, interestingly, the metabolite’s glucuronidation product was identified. We speculate that *m*/*z* 350.1023 was possibly completely glucuronidated in rats and led to no detection [25]. Deroussent et al. researched the metabolites and metabolic pathways of SAN in rat liver microsomes, and all of the identified metabolites were all included in this study. However, the reaction(s) involved in the metabolic pathways was different; Deroussent et al. reported that the metabolic process of SAN was oxidation, demethylation, and reduction, respectively, while this study suggests that reduction should be the first step, followed by oxidation, demethylation, hydroxylation, and glucuronidation [20]. To sum up, the metabolism of SAN in rats is more complex than that in vitro, and there are also different metabolic pathways; this needs to be further verified in further studies.

## 3. Materials and Methods

### 3.1. Chemicals and Reagents

Sanguinarine (purity > 98%) and dihydrosanguinarine (purity > 96%) were provided by Micolta Biresource Inc., Ltd. (Changsha, China). Formic acid was obtained from ROE. Acetonitrile and methyl alcohol were acquired from Merck. Sodium carboxymethylcellulose (CMC-Na, Lot: CC31154150) was purchased from Beijing Coolaber Technology Co., Ltd. (Beijing, China). Deionized water was purified using a Milli-Q system (Bedford, MA, USA). The other chemicals were of analytical grade and were commercially available.

### 3.2. Animals and Drug Administration

Male Sprague-Dawley (SD) rats (180–220 g) were purchased from Changsha Tianqin Biotechnology Co., Ltd. (Changsha, China), and were acclimatized for a week before any treatments. All of the experimental protocols were approved by the Animal Ethics Committee of Hunan Agricultural University, and the procedures were in accordance with the guidelines of the Committee on the Care and Use of Laboratory Animals of the U.S. National Institutes of Health. Twelve SD rats were randomly divided into four groups (three rats in each group), including one blank group and three experiment groups, which were used to gather plasma, urine, and feces, respectively. SAN (4 mg/mL) was suspended in 0.5% CMC-Na, which was given to the rats at a dose of 40 mg/kg through oral gavage.

### 3.3. Sample Collection and Pretreatment

#### 3.3.1. Sample Collection

The rats were housed in metabolism cages prior to drug administration. The plasma samples were collected using a retro-orbital puncture with a glass capillary from the fossa orbitalis vein 1, 2, and 4 h after dosing. The samples were collected into heparinized plastic tubes and centrifuged at 3000 rpm for 10 min, and the resulting plasma samples were merged. The urine and feces samples were gathered at 0–12, 12–24, 24–36, and 36–48 h after dosing and then merged, and the feces were air dried. All of the samples were stored at −80 °C until analysis. 

#### 3.3.2. Sample Pretreatment

The pretreatment methods for the plasma, urine, and feces were described in an article that has already been published [23]. First, 1 mL of the urine sample was mixed with an equal volume of acetonitrile for protein precipitation and vortexed for 2 min, and then centrifuged at 5000 r/min for 5 min, and the supernatant was collected and dried with nitrogen at 45 °C. The residue was reconstituted in 200 μL methanol and mixed for 2 min, then the sample was centrifuged at 12,000 r/min for 10 min and the supernatant was collected. The fecal sample (1 g) was ultrasonically extracted with 20 mL methanol for 30 min and then centrifuged at 5000 r/min for 5 min. The supernatant was collected and dried with nitrogen at 45 °C, the residue was dissolved using 200 μL methanol and mixed for 2 min, and then centrifuged at 12,000 r/min for 10 min and the supernatant was collected. Then, 250 μL of rat plasma was transferred into 1.5 mL tubes and 1 mL methanol was added and mixed for 2 min to precipitate the protein, the mixture was centrifuged at 12,000 r/min for 10 min. The supernatant was then collected to a clean tube and dried with nitrogen at 45 °C. The residue was reconstituted in 100 μL methanol and mixed for 2 min, and then the sample was centrifuged at 12,000 r/min for 10 min nd the supernatant was collected. Finally, an aliquot of 10 μL from all of the collected supernatants was injected into the UPLC-Q-TOF-MS/MS system for analysis.

### 3.4. Instruments and Analytical Conditions

A 1290 high-performance liquid chromatography system (Agilent Technologies, Palo Alto, CA, USA) equipped with a quaternary gradient pump, in-line degassing, and constant temperature column oven was used. SAN and its metabolites were separated on an ACQUITY UPLC TM HSS T_3_ column (100 mm × 2.1 mm, 1.8 μm) at a column temperature of 40 °C. The mobile phase, consisting of 0.1% formic acid in water (A) and 0.1% formic acid in acetonitrile (B), was delivered at a flow rate of 0.3 mL/min, and the injection volume was 1 µL. A linear gradient of 5–21% B for 0–9 min, 21–27% B for 9–11.5 min, 27–43% B for 11.5–15 min, 43–85% B for 15–28 min, 85–95% B for 28–30 min, 95% B for 30–33 min, and 5% B for 33.1–35 min was employed.

MS and MS/MS data were obtained in positive mode (*m*/*z*: 100–1000) using an electrospray ionization (ESI) interface on an Agilent Technologies 6530 Q-TOF. The parameters were as follows: drying gas temperature of 350 °C; drying gas flow of 12 L/min, nebulizer (N_2_) pressure of 35 psi, sheath gas (N_2_) temperature of 350 °C, sheath gas flow of 11 L/min, capillary voltage of 4000 V, nozzle voltage of 500 V, and fragmentor of 135 V. A collision energy of 20–40 V was used for the MS/MS analysis. An Agilent Masshunter B.08.00 was used for instrument control and data acquisition.

### 3.5. Data Analysis

Before the sample pretreatment and data acquisition, we summarized the metabolites and pathways of sanguinarine and other alkaloids (such as chelerythrine) by searching relative articles. Then, the metabolic reaction, pathway, and *m*/*z* of the possible metabolites of sanguinarine were concluded in an Excel, which was named a private database. After data acquisition and background correction, we searched for possible metabolites in total ion chromatogram using the database and selected the high abundance components that existed in the database. The MS/MS spectra of the selected substances were acquired using the target MS/MS method, then the structure of the component fragments of metabolites were inferred according to the fragmentation pattern of sanguinarine.

## 4. Conclusions

In this study, the metabolism of SAN was firstly explored in rats in vivo via UPLC-Q-TOF-MS/MS. Twelve metabolites were identified based on the chromatographic retention times, accurate mass measurements, MS/MS fragmentation patterns. and fragment ion mass. The metabolic pathways involved reduction, oxidation, demethylation, hydroxylation, and glucuronidation. This research have identified the metabolites of SAN firstly in rats, which is helpful to deeply research the metabolic mechanism and find out the key enzymes of SAN in rats. The mechanism of SAN is important to decrease the toxicity by interfering with drug-metabolizing key enzymes, and also ensure the safety and widespread use of *Macleaya cordata* (Willd.) R. Br.

## Figures and Tables

**Table 1:** Identification of SAN metabolites in rats using UPLC/Q-TOF-MS.

Met No.	RT (min)	Elemental Composition	Metabolic Pathway	Ionization	Calcd *m*/*z*	Meas *m*/*z*	Mass Error (ppm)	Plasma	Urine	Feces
M0	13.496	C_20_H_14_NO_4_	Parent	[M]^+^	332.0917	332.0913	3.31	√	√	√
M1	24.281	C_20_H_15_NO_4_	Reduction	[M+H]^+^	334.1074	334.1075	0.30	√	√	√
M2	19.73	C_20_H_17_NO_4_	Oxidation	[M+H]^+^	336.1230	336.1226	−1.19	√		√
M3	12.098	C_20_H_16_NO_4_	Oxidation	[M]^+^	334.1074	334.1072	−0.60			√
M4	11.014	C_19_H_14_NO_4_	Demethylation	[M]^+^	320.0917	320.0893	−7.50			√
M5	12.721	C_19_H_14_NO_4_	Demethylation	[M]^+^	320.0917	320.0912	−1.56			√
M6	16.749	C_19_H_15_NO_4_	Demethylation	[M+H]^+^	322.1074	322.1078	1.24			√
M7	19.32	C_20_H_14_NO_5_	Hydroxylation	[M]^+^	348.0866	348.0866	0.00		√	√
M8	17.864	C_20_H_16_NO_6_	Hydroxylation	[M]^+^	366.0972	366.0971	−0.27			√
M9	14.885	C_25_H_23_NO_10_	Glucuronidation	[M+H]^+^	498.1395	498.1378	3.41	√		
M10	23.847	C_26_H_24_NO_10_	Glucuronidation	[M]^+^	510.1395	510.1444	9.61			√
M11	15.213	C_26_H_25_NO_10_	Glucuronidation	[M+H]^+^	512.1551	512.1541	1.95		√	
M12	15.691	C_26_H_24_NO_11_	Glucuronidation	[M]^+^	526.1344	526.1377	6.27		√	

“√”: metabolites have been detected.

## Data Availability

Data are contained within the article and Supplementary Materials.

## Supplementary Materials

The following supporting information can be downloaded at: https://www.mdpi.com/article/10.3390/molecules28227641/s1, Figure S1: Structures of SAN and its metabolites.
