# Supplementary figures and images for: Identification of Sanguinarine Metabolites in Rats Using UPLC-Q-TOF-MS/MS
Source: Molecules. 2023 Nov 17;28(22):7641. doi: 10.3390/molecules28227641 (PMC10674372; doi:10.3390/molecules28227641)

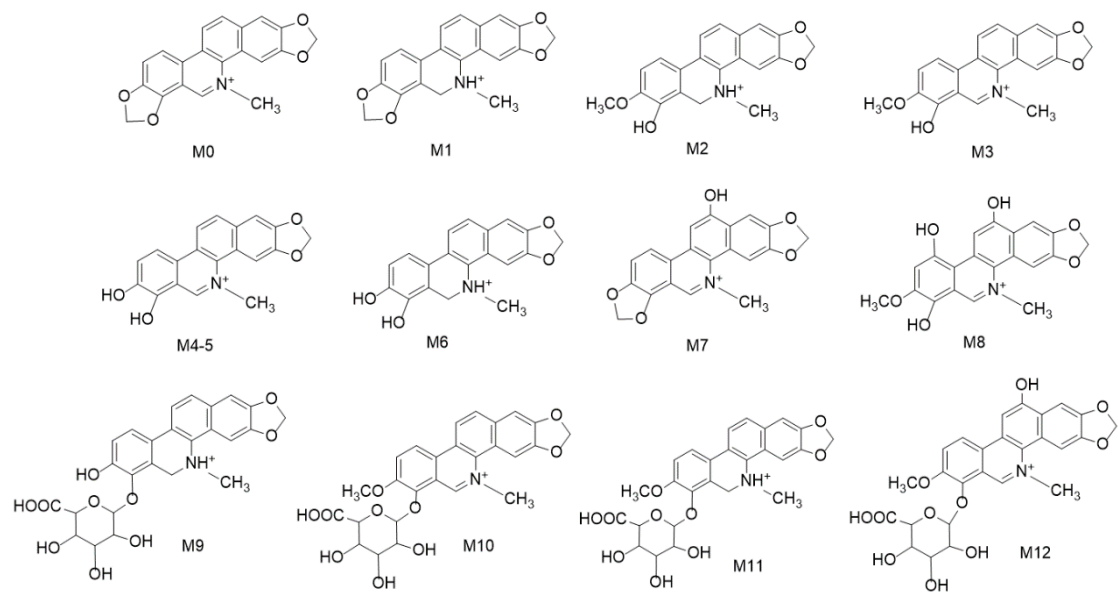

Figure S1. Structures of SAN and its metabolites

Supplement: Supplementary file 1 [file molecules-28-07641-s001.zip › molecules-2684746-supplementary.pdf]
